# Complete genome sequence of *Lactiplantibacillus plantarum* SE1, isolated from the Japanese fermented pickle “Pesora-zuke”

**DOI:** 10.1128/mra.01225-25

**Published:** 2026-03-05

**Authors:** Toshihiro Cho, Toshitaka Kumagai, Keietsu Abe, Jun Kaneko

**Affiliations:** 1 Department of Regional Resources Development, Yamagata Research Institute of Technology 230171, Yamagata, Japan; 2 Laboratory of Applied Microbiology, Department of Agricultural Chemistry, Graduate School of Agricultural Science, Tohoku University 13101, Sendai, Japan; 3 Fermlab Inc., Tokyo, Japan; 4 Laboratory of Fermentation Microbiology, Department of Agricultural Chemistry, Graduate School of Agricultural Science, Tohoku University 13101, Sendai, Japan; Queens College Department of Biology, Queens, New York, USA

**Keywords:** *Lactiplantibacillus plantarum*, genome sequencing, exopolysaccharide biosynthesis, fermented food isolate

## Abstract

We report the complete genome sequence of *Lactiplantibacillus plantarum* SE1, isolated from the traditional Japanese fermented pickled eggplant, “Pesora-zuke”. The genome consists of one chromosome and four plasmids. Four predicted exopolysaccharide (EPS) biosynthetic gene clusters were identified, one of which was plasmid-encoded.

## ANNOUNCEMENT

*Lactiplantibacillus plantarum* is widely used in food fermentation and probiotic research (1–3). This species exhibits high plasmid content and genomic diversity, characterized by multiple exopolysaccharide (EPS) biosynthesis gene clusters for adaptation (4, 5). The proportion of chromosomally and plasmid-encoded EPS gene clusters may indicate the potentially unique contribution of plasmids in EPS biosynthesis and diversity (6). Strain SE1 was isolated in 2019 from lactic acid bacteria populations in the brine of Pesora-zuke, a traditional fermented pickled eggplant from Yamagata Prefecture, Japan, based on yellow colony formation on BCP-supplemented plate count agar. Strain SE1 was selected for its high-viscosity phenotype by producing abundant EPS on De Man–Rogosa–Sharpe (MRS) agar. Cells grown in MRS medium were identified as *Lactiplantibacillus plantarum* by MALDI-TOF MS (Bruker) after ethanol–formic acid extraction (7, 8).

A single colony of SE1 was cultured in MRS broth at 35°C for 48 h, and cells were then submitted to Seibutsu Giken (Kanagawa, Japan) for PacBio sequencing. Genomic DNA was extracted using the Genomic-tip 20G kit (QIAGEN). Quality and quantity were assessed using the Agilent HS Genomic DNA 50 kb Kit on a 5200 Fragment Analyzer System (Agilent Technologies) and QuantiFluor dsDNA System and Quantus Fluorometer (Promega). DNA was treated with the Short Read Eliminator XS (PacBio) to remove short fragments and sheared to approximately 10-20 kb using a g-TUBE (Covaris). The library was prepared using the SMRTbell Express Template Prep Kit 2.0 (PacBio), and polymerase complexes were formed using the Binding Kit 2.2 (PacBio) (9). Sequencing was performed on a Sequel IIe system (PacBio), yielding 25,076 long reads (218,596,681 bp; mean length of 8.72 kb). Subreads were generated after adapter removal using SMRT Link v.10.1.0.119528 (9, 10). Circular consensus sequences (CCS) reads with quality values <20 were filtered using Filtlong v0.2.0 to obtain high-fidelity (HiFi) reads (10, 11). HiFi reads with quality scores >20 (21,563 reads) were assembled using Flye v2.9.1 with default parameters (12). Genome coverage was 63.6×, with an N50 of 3,287,249 bp corresponding to the complete circular chromosome. Chromosome and plasmid contiguity and circularity were evaluated using Bandage v0.8.1 (13). The completeness and contamination of the assembly were assessed using CheckM v1.2.0 (14), yielding 99.07% completeness and 2.78% contamination. Annotation was performed using DFAST v1.6.0 (15).

The genome of strain SE1 consists of a circular chromosome (3,287,249 bp) and four circular plasmids (69,692; 43,208; 26,101; and 9,152 bp), totaling 3,435,402 bp (44.29% GC content) (Table 1). Genome completeness was confirmed by BUSCO (100%; 402/402 Lactobacillales_odb10 data set) (16). GC skew analysis showed a single-inflection pattern indicating a circular chromosome. Average nucleotide identity (ANI) analysis using fastANI (17) showed 98.9% identity with *L. plantarum* WCFS1, exceeding the 95–96% species threshold. Default parameters were used for all software unless noted.

**TABLE 1:** Whole-genome sequencing overview of SE1

Genetic element	Size(bp)	Coding genes	tRNAs	rRNAs
Chromosome	3,287,249	3,093	68	16
Plasmid 1	69,692	70	0	0
Plasmid 2	43,208	43	0	0
Plasmid 3	26,101	28	0	0
Plasmid 4	9,152	10	0	0
Total	3,435,402	3,244	68	16

Notably, plasmid 3 of SE1 harbored a predicted EPS biosynthesis gene cluster, the gene organization of which differed from that of the EPS clusters in other reported strains (6, 18, 19). The genome information of SE1 provides a resource on the role of plasmids in EPS biosynthesis in *L. plantarum*.

## Data Availability

This Wholewhole genome shotgun project has been deposited at DDBJ/ENA/GenBank under accession numbers BAAHZD010000001 to BAAHZD010000005, BioSample SAMD00900825, and BioProject PRJDB20628.
